# Establishment of a transgenic mouse model with liver-specific expression of secretory immunoglobulin D

**DOI:** 10.1007/s11427-012-4301-3

**Published:** 2012-04-14

**Authors:** Ping Wang, ZhiGuo Wei, BoWen Yan, Tan Huang, KeMian Gou, YunPing Dai, Min Zheng, MeiLi Wang, XueQian Cheng, XiFeng Wang, Chen Xu, Yi Sun

**Affiliations:** 1 College of Biological Sciences, China Agricultural University, State Key Laboratories for Agrobiotechnology, Beijing, 100193 China; 2 College of Animal Science & Technology, Henan University of Science and Technology, Henan, 471003 China

**Keywords:** sIgD, liver-specific expression vector, HIDS, MVK

## Abstract

Mutation of mevalonate kinase (MVK) is thought to account for most cases of hyperimmunoglobulinemia D syndrome (HIDS) with recurrent fever. However, its mechanism and the relationship between elevated serum immunoglobulin D (IgD) and the clinical features of HIDS are unclear. In this study, we generated by fusion PCR a vector to express high levels of chimeric secretory IgD (csIgD) specifically in the liver. We then generated seven founder lines of transgenic mice by co-microinjection, and verified them using genomic PCR and Southern blotting. We detected the expression of csIgD by reverse transcription PCR, quantitative PCR, western blotting, and enzyme-linked immunosorbent assays. We demonstrated that csIgD could be specifically and stably expressed in the liver. We used flow cytometry to show that overexpression of csIgD in the bone marrow and spleen cells had no effect on B cell development. Morphologic and anatomical observation of the transgenic mice revealed skin damage, hepatosplenomegaly, and nephromegaly in some transgenic mice; in these mice, pathological sections showed high levels of cell necrosis and protein-like sediments in the liver, spleen, and kidney. We demonstrated that the genomic insertion sites of the transgenes did not disrupt the *MVK* gene on mouse chromosome 5. This transgenic mouse will be useful to explore the pathogenesis of HIDS.

Hyperimmunoglobulinemia D syndrome (HIDS), also called receptor-associated periodic syndrome or etiocholanolone fever, is characterized by high serum levels of immunoglobulin D (IgD) and recurrent febrile attacks, and may also include arthritis, lymphadenopathy, hepatosplenomegaly, and skin rash [1–3]. It was originally described in patients of Dutch ancestry by Van der Meer *et al*. in 1984 [4] but has now been reported all over the world, including in Japan [5,6]. Its pathogenesis is unknown and there are no effective therapies. High levels of IgA and tumor necrosis factor alpha (TNF-α) are found in the sera of some HIDS patients [7]. Patients with HIDS also secrete large amounts of the proinflammatory cytokine interleukin 1β (IL-1β), which may be connected with low levels of isoprenoid metabolic end products caused by a lack of mevalonate kinase (MVK) [8].

In 1999, Drenth *et al*. [9] obtained samples from 34 patients and 44 unaffected members from 16 families with HIDS who originated from the Netherlands (8), France (5), the United Kingdom (1), Spain (1), and the Czech Republic (1). An *MVK* gene missense mutation resulting in decreased MVK activity was identified. Reduced MVK activity in fibroblasts suggested that the *MVK* mutation caused HIDS. Since then, other *MVK* gene mutations or deletions have been found in some HIDS patients [10,11]. MVK is a protease in the cytoplasm that participates in the synthesis of cholesterol and non-sterol isoprenoid. Human *MVK* is located on chromosome 12q24. The V377I mutation in *MVK* occurs homozygously in approximately 20% of HIDS patients and heterozygously in most of the others. The second most common mutation is I268T [12,13].

The effect of deletion of a single *Mvk* allele in mice was reported by Hanger et al. in 2007 [14]. These authors reported significantly increased IgD levels, and a trend towards higher IgA and TNF-α levels, in $Mvk^{+/-}$ sera compared with $Mvk^{+/+}$ littermate sera, which was accompanied by periodic fever and hepatosplenomegaly. Moreover, Ammouri *et al*. [15] reported that, among fifty HIDS patients, 38 had high serum IgD, even though only 19 were homozygotes or composite heterozygotes for *MVK* mutations. This indicates that secretory IgD (sIgD) plays an important role in the clinical presentation of HIDS. The function of sIgD is unclear, although it has been reported to combine with antigens of microorganisms and their products. In early inflammatory responses in patients with contact dermatitis, sIgD could induce bone marrow cells—mainly neutrophils, eosinophils, and basophils—to migrate to the skin [16]. sIgD binds to basophils through a calcium-mobilizing receptor that induces antimicrobial and B cell-stimulating factors, after IgD cross-linking. sIgD is an important immune-conditioning molecule; its interaction with its binding partners induces cell activation, organization penetration, immune activation factor release, and enhances other immune defenses. However, excess activation can lead to inflammation and tissue destruction [17]; the effects and levels of increased sIgD vary among HIDS patients [18].

The relationships among sIgD, MVK, and the inflammatory response are unclear; and it is unknown how increased sIgD or *MVK* mutations could cause inflammation. To explore this question, we constructed a transgenic mouse model with liver-specific sIgD overexpression.

### 1 Materials and methods

#### 1.1 Construction of vectors

##### *1.1.1 Fusion PCR to generate the en-pAlb fragment*

Genomic DNA was extracted from the liver tissue of Kunming white mice (purchased from the Zoology Department of the National Family Planning Institute, Beijing, China). The 1.97-kb albumin (*Alb*) enhancer [19] and the 412-bp *Alb* promoter [20,21] were amplified by PCR. The primers for the mouse *Alb* enhancer were en-Alb-F (5′-AAA AAC GCG TAT TTG AAT ACA CAC TTT TGT CAT G-3′) and en-Alb-R (5′-AGG TTG TTC ATC CCA AAG TTA TCA ATC AAA GCA G-3′). The primers for the mouse *Alb* promoter were p-Alb-F (5′-CTG CTT TGA TTG ATA ACT TTG GGA TGA ACA ACC T-3′) and p-Alb-R (5′-AAA AGC TAG CTG CCA GAG GCT AGT GGG GTT G-3′). The underlined sequences indicate *Mlu* I and *Nhe* I sites, respectively. Four adenine nucleotides were used to protect the restriction enzyme site. The en-Alb-F and p-Alb-R primers were then used to amplify the 2.35-kb *en-pAlb* fragment by fusion PCR.

##### *1.1.2 Transgene amplification*

Total RNA was isolated using Trizol (Tiangen, Beijing, China) from spleen tissue of Kunming white mice; 1 μg of total RNA was used for first-strand cDNA synthesis with M-MLV Reverse Transcriptase (Promega, Madison, WI). The coding regions of the sIgD heavy chain constant region (IgDHC) and kappa chain constant region (KC) were amplified from cDNA. The 803-bp *IgDHC* region was amplified using primers IgDHC-F (5′-CTG GTC ACC GTC TCC TCA GGT GAT AAA AAG GAA CCT GAC-3′) and IgDHC-R (5′-TTT TGC GGC CGC TCA GGC AAG GGC AGG ACC A-3′). The 348-bp KC region was amplified using primers KC-F (5′-CAA GCT CGA GAT CAA ACG GGC TGA TGC TGC ACC AAC-3′) and KC-R (5′-TTT TGC GGC CGC CTA ACA CTC ATT CCT GTT G-3′). The underlined sequences indicate a *Not* I site. Four thymidines were used to protect the restriction enzyme site.

The coding regions of the heavy chain variable region (IgGHV) and kappa chain variable region (KV) of a human IgG1 monoclonal antibody against Hepatitis A virus (HAV) were amplified by PCR from plasmids *pHAVH3* and *pHAVL3* (saved by our laboratory). The 450-bp *IgGHV* region was amplified using primers IgGHV-F (5′-TTT TGA ATT C**AG CAT GG**G TGA CAA TGA CAT CCA C-3′) and IgGHV-R (5′-GTT CCT TTT TAT CAC CTG AGG AGA CGG TGA CC-3′). The underlined sequence indicates an *Eco*R I site; the sequence in bold typeface shows the Kozak fragment. Four thymidines were used to protect the restriction enzyme site. The 410-bp KV region was amplified using primers IgGHV-F (as above) and KV-R (5′-GTT GGT GCA GCA TCA GCC CGT TTG ATC TCG AGC TTG-3′).

Chimeric (c) *sIgD-H* (1214 bp) and *cKappa* (722 bp) were then amplified by fusion PCR. The primers for *csIgD-H* were IgGHV-F and IgDHC-R; the primers for *cKappa* were IgGHV-F and KC-R.

##### *1.1.3 Construction of the en-pAlb-csIgD-H and en-pAlb-cKappa vectors*

*csIgD-H* and *cKappa* were separately cloned into the expression plasmid pcDNA3.1(+) and digested with *Eco*R I and *Not* I. Using *Mlu* I and *Nhe* I restriction enzyme digestion of these two expression vectors, the *CMV* promoter was replaced with *en-pALB*. The resulting plasmids, designated en-pAlb-csIgD-H and en-pAlb-cKappa, were under control of the mouse *Alb* regulatory sequences to direct gene expression specifically in the liver.

### 1.2 Generation of transgenic mice

The 4059-bp *csIgD-H* fragment and the 3567-bp *cKappa* fragment were released from their plasmids by *Mlu* I and *Nae* I digestion, respectively. Each fragment contained the mouse *en-pAlb*, a T7 promoter, an Ig secretory leader, either the *csIgD-H* gene or the *cKappa* gene, followed by the bovine growth hormone (BGH) poly(A) (Figure 1). The two fragments were purified after agarose gel electrophoresis (Omega, Boston, Massachusetts) and subsequently co-microinjected at a 1:1 molar ratio into the pronuclei of fertilized Kunming white ova according to standard protocols. Genomic DNA was extracted from mouse tail biopsies. The presence of the transgenes was verified by PCR with primers p-Alb-F (as above) and en-pAlb-R (5′-AGG AAG GGA AGA AAG CGA AAG-3′). The primers were specific to the *Alb* promoter and BGH poly(A) sequences, respectively, and so could detect both the heavy and light chain genes simultaneously. Genomic DNA (10 μg) was digested with *Dra* I and analyzed by Southern blotting with digoxigenin-labeled hybridization probes. The probes were the amplified products of the *csIgD-H* and *cKappa* genes.

### 1.3 Reverse transcription PCR (RT-PCR) and quantitative PCR (Q-PCR)

Total RNA was extracted using Trizol (CwBio, Beijing, China) according to the manufacturer's instructions from the liver tissues of mice from different families. The RNA concentration was determined using a Nanodrop 2000 (Thermo Scientific, USA); 2 μg was used for first-strand cDNA synthesis by M-MLV Reverse Transcriptase (Promega, Madison, WI). The primers used to detect chimeric mRNA expression were HV-HC-F (5′-GTC AGG TAC AAC TAT GGT GTC-3′) and HV-HC-R (5′-CAA GTG TGG TTG AGG TTC AGT-3′), KV-KC-F (5′-TTC CTG ACC GAT TCT CTG GCT-3′) and KV-KC-R (5′-TCA AGA AGC ACA CGA CTG AGG-3′), of which the forward primer and reverse primer were complementary to the variable region and constant region, respectively, of *csIgD-H* or *cKappa*, amplifying 308 and 243 bp fragments, respectively. Mouse *β-actin* was used as an internal control. The primers for mouse *β-actin* were β-actin-F (5′-GCT GTA TTC CCC TCC ATC GT-3′) and β-actin-R (5′-GGA TAC CTC TCT TGC TCT GG-3′), amplifying a 109 bp fragment.

Q-PCR was performed using SYBR green super mix (#4334973; Applied Biosystems, Foster City, CA) on a 7900 Q-PCR machine (Applied Biosystems). The data were analyzed in Excel (Microsoft Office Excel 2007) using the $2^{-\Delta\Delta C_t}$ relative quantification method. The primers for *csIgD-H* and *cKappa* were HC-F (5′-GTG TCC AAT ACT ACT TTG ACT ACT-3′) and HC-R (5′-CAT TTT CCT CTG GGG CTT TGC-3′); and KC-F (5′-TAT TCG GCG GAG GGA CCA AG-3′) and KC-R (5′-CAA GAA GCA CAC GAC TGA GG-3′), respectively. The primers for *β-actin* were β-actin-F and β-actin-R (as above).

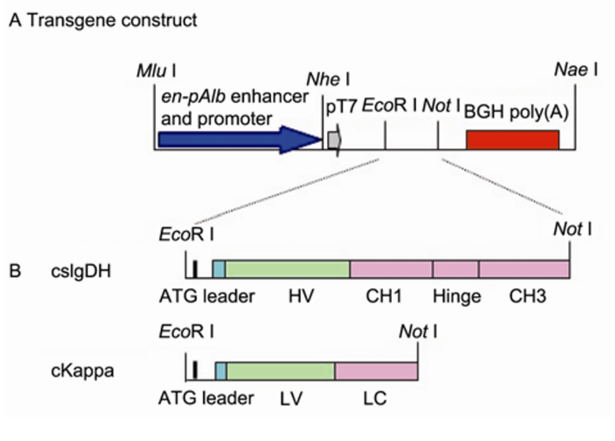

**Figure 1** Schematic of the *csIgD-H* and *cKappa* transgene constructs used for microinjection. A, Structure of the transgene construct released from the en-p-csIgD-H/cKappa vector with *Mlu* I/*Nae* I, respectively. The transgene backbone contains the *en-pAlb* enhancer and promoter, a T7 promoter, and BGH poly(A). Dotted lines indicate the site of insertion of the *csIgD-H* and *cKappa* sequences between the *Eco*R I and *Not* I restriction sites. B, Blue, green, and pink boxes represent the Ig secretory leader, variable regions (VH and VL), and constant regions (HC and KC), respectively. Exons encoding the constant regions are indicated as CH1, Hinge, and CH3 for *csIgDH* and as LC for *cKappa*. The translational start codons are indicated as ATG. Relevant restriction enzymes sites are shown.

### 1.4 SDS-polyacrylamide gel electrophoresis (PAGE) and Western blotting

Blood from the orbital sinus of transgenic and non-transgenic mice was collected in 0.5-mL centrifuge tubes and stored overnight at 4°C. Serum was obtained by centrifugation at 1200×*g* for 15 min at 4°C, and stored at −80°C. The total serum protein concentration was determined by the BCA method (Beyotime, China). Serum samples containing 8 μg of protein were mixed with 5×SDS, denatured for 5 min at 95°C, and then separated on 10% SDS-PAGE gels under reducing conditions, using separate samples for staining with Coomassie brilliant blue and Western blotting. The separated proteins were electrophoretically transferred to nitrocellulose membranes (Amersham Pharmacia, UK), washed twice in Tris-buffered saline/20% Tween 20 (TBST) for 5 min, incubated overnight in blocking buffer (5% defatted milk in TBST) at 4°C, followed by two washes in TBST for 10 min each. The membranes were then incubated with a 1:1000 dilution (in 0.5% blocking reagent) of horseradish peroxidase-conjugated monoclonal anti-mouse IgD antibody (eBioscience, San Diego, California) for 1 h at room temperature, washed three times for 10 min each, and then visualized using enhanced chemiluminescence reagents

(Amersham Biosciences, UK) according to the manufacturer's instructions. Mouse *GAPDH* was used as an internal control when we detected the sIgD expression in transgenic mouse liver with Western blotting.

#### 1.5 Enzyme-linked immunosorbent assay (ELISA)

A mouse serum IgD-ELISA kit (BlueGene, China) was brought to room temperature for 30 min before use, according to the manufacturer's instructions. One hundred microliters of serum standard, sample, or distilled water (blanks) was added to each well of a 96-well microtiter plate; 50 μL of enzyme-linked marker solution was then added to each well except the blank wells, the wells were covered with adhesive strip and incubated for 1 h at 37°C. The microtiter plate was then washed five times, and patted dry with absorbent paper. Next, 50 μL of chromogenic reagents A and B were added to each well except the blank wells, and the plate was incubated in the dark for 15 min at 20–25°C, followed by the addition of 50 μL of stop solution to each well. The optical density at 450 nm of each well was determined within 30 min, using an automatic microplate reader (Bio-rad Co., Philadelphia, USA).

#### 1.6 Analysis of B cell development by flow cytometry

For the isolation of bone marrow cells and spleen cells, transgenic mice and sex-matched littermate non-transgenic mice were sacrificed by cervical dislocation. The flesh of the mouse legs was stripped, and then 2 mL syringes filled with phosphate-buffered saline (PBS) were inserted into the bone cavities to flush the bone marrow into 50-mL centrifuge tubes that were kept on ice. Spleen tissues were placed onto a 200-mesh sieve (Nanjing Xiaoxiao Instrument and Equipment Co., Ltd.), sprinkled with a small amount of PBS, and gently broken into intact single cells with the core of a syringe; the cells were then flushed with PBS into 50-mL centrifuge tubes kept on ice. Two volumes of red blood cell lysis buffer (eBioscience, San Diego, California) were added to remove red blood cells, mixed and incubated on ice for 10 min. The reaction was then terminated by adding five volumes of PBS. The single-cell suspensions of bone marrow and spleen were centrifuged at 300×*g* for 10 min at 4°C. The supernatant was discarded and the cells resuspended in an appropriate amount of PBS. Bone marrow and spleen cells were then counted using a blood cell count board.

The cells were divided into five fractions for the addition of antibody: blank (no antibody added); phycoerythrin-conjugated Cy5.5 anti-mouse B220; fluorescein isothiocyanate-conjugated anti-mouse IgM; phycoerythrin-conjugated anti-mouse CD43; and all three of the above antibodies. The dilution and dosage of antibodies were according to the manufacturer's instructions (eBioscience, San Diego, California). These ten tubes of cells (five each for bone marrow and spleen) were incubated in the dark for 20 min at 4°C, according to the manufacturer's instructions. The cells were then washed twice with PBS, centrifuged at 300×*g* for 10 min at 4°C, resuspended in 150 μL PBS and kept on ice. The B cells were analyzed by flow cytometry (MOFLO High Performance Cell Sorter, Dako; Fort Collins, Colorado).

#### 1.7 Thermal asymmetric interlaced PCR (TAIL-PCR)

Three transgene-specific primers (tgp1: 5′-GAG CAG CAC CCT CAC ATT-3′ or 5′-CAG CAA GAG CCT AGC AAT-3′; tgp2: 5′-TGC CAG CCA TCT GTT GTT-3′; and tgp3: 5′-TCT GAG GCG GAA AGA ACC-3′) and an arbitrary degenerate primer (5′-NGT CGA SWG ANA WGA A-3′, 5′-AGW GNA GWA NCA WAG G-3′, or 5′-WGT GNA GWA NCA NAG A-3′) were used for TAIL-PCR. The primary PCR reaction contained 0.5–1.0 μg of genomic DNA. In the secondary or tertiary amplification, 0.65 μL of the first or secondary products was used as the template, respectively. The products of tertiary TAIL-PCR were separated on 1.5% agarose gels. A single band in each mouse sample was purified with a TIANgel Midi Purification Kit (Tiangen, China), sequenced, and analyzed by BLAST on the National Center for Biotechnology Information website. In addition, the morphology and anatomy observation and tissue sections (stained hematoxylin-eosin) analysis of the transgenic mice was detected.

### 2 Results

#### 2.1 Identification of the transgenic mice

Nine transgenic founders (Nos. 3, 4, 5, 6, 7, 12, 15, 24, and 37) were obtained from 37 mice analyzed by PCR (Figure 2A). All carried both the *csIgD-H* and *cKappa* transgenes. In addition, there were another three founders that carried either the *csIgD-H* or *cKappa* transgenes; these were not analyzed further. Southern blotting was used to confirm that both transgenes were integrated into the genome of all nine transgenic mice (Figure 2B). Probes specific for the 1214-bp *csIgD-H* and 722-bp *cKappa* fragments were mixed together, allowing both transgenes to be detected simultaneously. Of the founders, the founder 12 died and the founder 15 could not be passaged because of illness; however, 7 were mated with wild-type mice, all of which transmitted the transgenes to their offspring; of these, 26 $F_1$ transgenic mice with double transgenes were identified among 64 offspring.

#### 2.2 Expression of csIgD in the liver tissues of transgenic mice

Transgene mRNA expression in the liver tissues of transgenic mice was analyzed by RT-PCR (Figure 2C). Both the *csIgD-H* and *cKappa* transgenes were expressed in eight transgenic lines (Nos. 15 and 37 of the $F_0$ generation and six mice of the $F_1$ generation). There were no bands in the negative control mice; a band for the housekeeping gene *β-actin* was visible in each at ~109 bp. This confirmed that *csIgD-H* was expressed from the chimeric mRNA in the liver tissues of all transgenic mice. Quantitative PCR showed differing levels of expression between *csIgD-H* and *cKappa* in individual mice and among different mice (Figure 2D).

Serum samples were collected from five transgenic founder mice or $F_1$ mice ($F_0$6–$F_1$7, $F_0$15, $F_0$7–$F_1$24, $F_0$37, and $F_0$4–$F_1$50) that carried both transgenes. The expression of serum sIgD was analyzed by Western blotting. A single band was detected for sIgD-H under reducing conditions with the expected molecular weight of 46 kD (Figure 3A). The expression of sIgD in the liver and spleen of $F_0$37 and two negative littermates was then analyzed. A single 46-kD

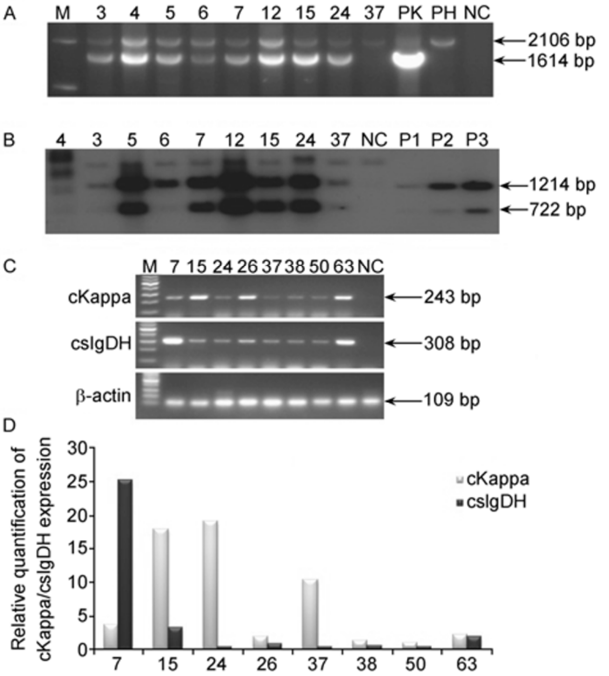

**Figure 2** Identification of transgenic founder lines. A, PCR detection of transgenic founders. M, 1 kb DNA ladder; PK, plasmid positive control for *cKappa*; PH, plasmid positive control for *csIgD-H*; NC, negative control; transgenic founders numbered 3, 4, 5, 6, 7, 12, 15, 24 and 37. Amplified products: 2106 bp (*csIgD-H*) and 1614 bp (*cKappa*). B, Southern blotting of transgenes. Digested genomic DNA was hybridized with mixed probes for amplified *csIgD-H* and *cKappa* fragments; hybridization signals for *csIgD-H* and *cKappa* are indicated that the sizes correspond to transgenes. NC, genomic non-transgenic mouse DNA negative control; P1, P3, P5, positive plasmid DNA equivalents for 1, 3, and 5 gene copies, respectively. C, RT-PCR analysis of liver transgene expression. M, 100 bp DNA marker ladder; transgenic mice (came from different transgenic founder or their offspring), $F_0$6–$F_1$7, $F_0$15, $F_0$7–$F_1$24, $F_0$3–$F_1$26, $F_0$37, $F_0$5–$F_1$38, $F_0$4–$F_1$50, and $F_0$24–$F_1$63; NC, non-transgenic mouse as negative control. The mouse *β-actin* gene was used as an internal control. D, Q-PCR analysis of the liver transgene expression of eight transgenic mice.

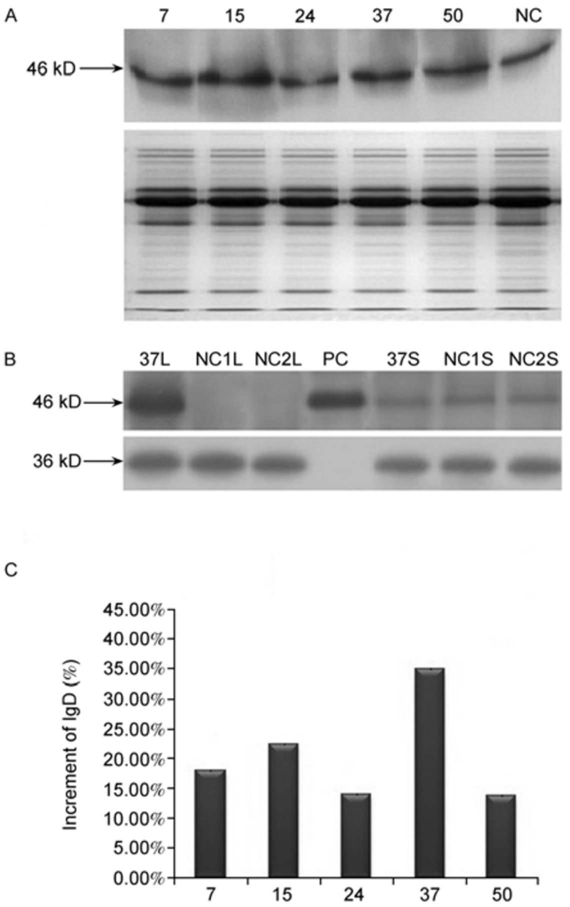

**Figure 3** Detection of sIgD by Western blotting and ELISA. A, Western blot of sIgD in transgenic mouse serum. Top panel, Western blot for sIgD in five transgenic mice; NC, negative control; bottom panel, corresponding polyacrylamide gel stained with Coomassie brilliant blue. B, Western blotting of liver (L) and spleen (S) of transgenic mouse $F_0$37 and two non-transgenic negative controls (NC1 and NC2). The positive control (PC) is serum from transgenic mouse $F_0$37. Top panel, sIgD; bottom panel, GAPDH. C, ELISAs for five transgenic mice. The vertical axis indicates the percentage increase in IgD in the serum of five transgenic mice compared with the average sIgD level in seven age-matched non-transgenic mice.

band was detected in both tissues of mouse $F_0$37; in controls, bands were observed in the spleen but not the liver (sIgD always expressed in spleen but no liver in normal condition) (Figure 3B). By ELISA, the serum sIgD level in each transgenic line was greater than that in control mice (Figure 3C).

### 2.3 B cell development in transgenic mice

The bone marrow and spleen cells from a randomly selected transgenic mouse ($F_0$4–$F_1$81) and a sex-matched littermate negative control were analyzed by flow cytometry. There were no significant differences between them in the content of pro-B ($B220^+CD43^+IgM^-$), pre-B ($B220^+CD43^-IgM^-$), or immature B ($B220^+CD43^-IgM^+$) bone marrow cells or mature spleen B cells ($B220^+IgD^+IgM^{+/-}$) (Figure 4). This in-

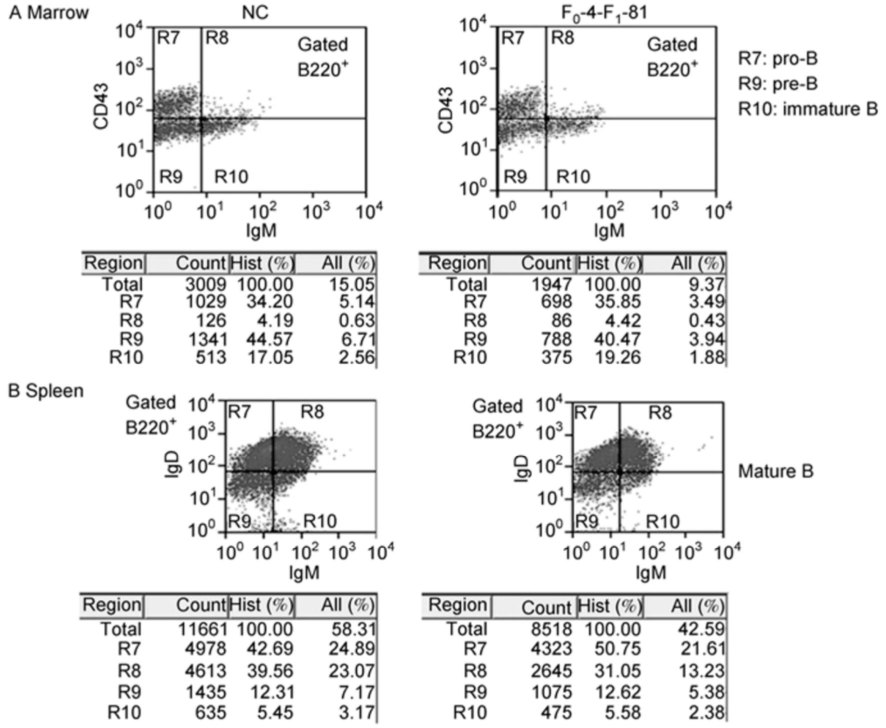

A Marrow, NC:

| Region | Count | Hist (%) | All (%) |
|---|---|---|---|
| Total | 3009 | 100.00 | 15.05 |
| R7 | 1029 | 34.20 | 5.14 |
| R8 | 126 | 4.19 | 0.63 |
| R9 | 1341 | 44.57 | 6.71 |
| R10 | 513 | 17.05 | 2.56 |

A Marrow, $F_0$-4-$F_1$-81:

| Region | Count | Hist (%) | All (%) |
|---|---|---|---|
| Total | 1947 | 100.00 | 9.37 |
| R7 | 698 | 35.85 | 3.49 |
| R8 | 86 | 4.42 | 0.43 |
| R9 | 788 | 40.47 | 3.94 |
| R10 | 375 | 19.26 | 1.88 |

B Spleen, NC:

| Region | Count | Hist (%) | All (%) |
|---|---|---|---|
| Total | 11661 | 100.00 | 58.31 |
| R7 | 4978 | 42.69 | 24.89 |
| R8 | 4613 | 39.56 | 23.07 |
| R9 | 1435 | 12.31 | 7.17 |
| R10 | 635 | 5.45 | 3.17 |

B Spleen, $F_0$-4-$F_1$-81:

| Region | Count | Hist (%) | All (%) |
|---|---|---|---|
| Total | 8518 | 100.00 | 42.59 |
| R7 | 4323 | 50.75 | 21.61 |
| R8 | 2645 | 31.05 | 13.23 |
| R9 | 1075 | 12.62 | 5.38 |
| R10 | 475 | 5.58 | 2.38 |

**Figure 4** Fluorescence-activated cell sorting analysis of bone marrow and spleen cells from a transgenic ($F_0$4–$F_1$81) and a non-transgenic (NC) mouse. A, Bone marrow B cells (pro-B, pre-B, and immature B). B, Spleen B cells (mature B).

dicates that sIgD overexpression in transgenic mice has no effect on B cell development.

### 2.4 Integration sites of the transgenes

We used genome walking (TAIL-PCR) to detect the site of integration of the transgene fragments in three transgenic mice ($F_0$6–$F_1$7, $F_0$7–$F_1$18, and $F_0$4–$F_1$50) (data not shown). The transgenes in these three mice integrated into chromosomes 14, 13, and 1, respectively; there were no functional genes near the insertion sites. This indicates that the *Mvk* locus on mouse chromosome 5 was not disrupted by the transgenes.

### 2.5 The morphology and anatomy observation of the transgenic mice

In observing the morphology and anatomy of four of the transgenic lines ($F_0$15, $F_0$7, $F_0$4, and $F_0$37), we found that these mice had skin ulcers, hepatomegaly, splenomegaly, and renomegaly, with livers 2.5–3.3 times, spleens 4.4–6 times, and kidneys 2.7–3.3 times the size of those of non-transgenic littermates (Figure 5A). Hematoxylin-eosin staining of tissue sections showed that these organs were diseased, with a high degree of cell necrosis and fiber-like protein precipitation (Figure 5B). These four transgenic founders were mated with wild type mice. $F_0$15 and $F_0$37 could not be passaged because of illness; $F_0$7 had two positive offspring, one of which had skin ulcers, hepatosplenomegaly, and renomegaly, as did two of the three positive offspring from $F_0$4.

## 3 Discussion sIgD was once considered to have no function, considering its low levels in serum and short half-life. Recent reports of HIDS [22–25] have caused researchers to re-examine sIgD, but its specific role in HIDS is still not clear. To facilitate this research, we generated a mouse model that stably and efficiently expresses sIgD. In this study, we found that csIgD could be expressed in the liver of mice under the control of the *Alb* enhancer and promoter. The mouse *Alb* promoter contains six *cis*-acting elements [20], which bind hepatocyte nuclear factor 1, CCAAT-binding protein, D-binding protein, leucine chlorolactamase, nuclear factor Y, and NF-1, respectively, and thus initiate gene transcription in the liver [26,27]. In addition, enhancers located 10.4–8.5 kb upstream can increase promoter activity 50-fold [28–30]. The sIgD expression was increased in different degrees in individual mice. Most mice carried both transgenes and most transgenic founders transmitted both

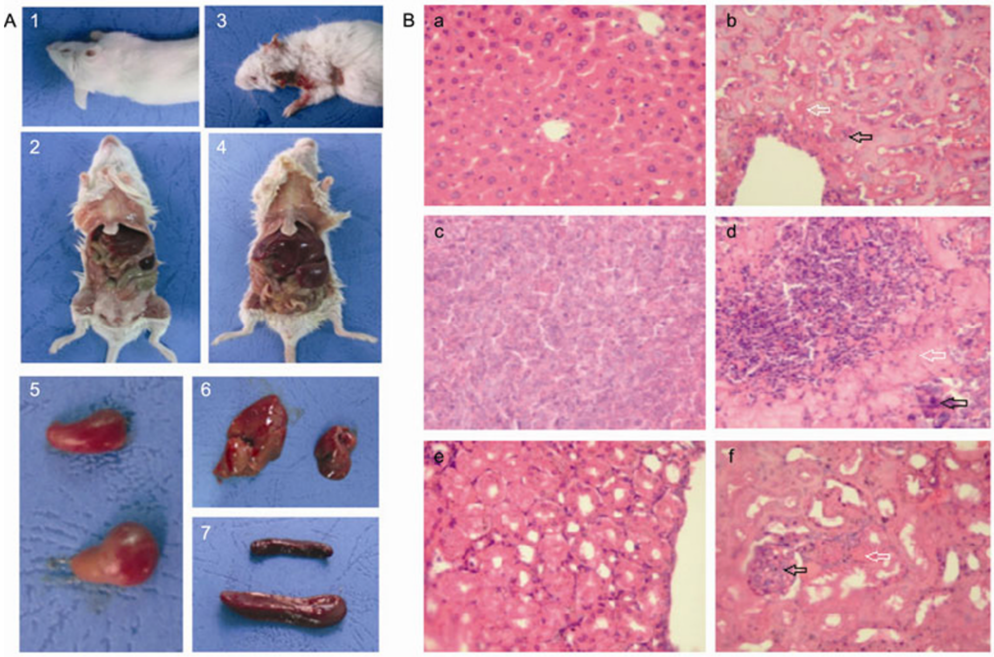

**Figure 5** Gross and microscopic observation of affected transgenic mice and negative controls. A, Morphology and anatomy of affected transgenic mice. 1 and 2, control mouse; 3 and 4, affected transgenic mouse; 5–7, kidney, liver and spleen, respectively, of control and affected transgenic mouse; in each case, the larger organ came from the transgenic mouse. B, Representative hematoxylin and eosin-stained tissue sections of diseased organs. a, c, and e, liver, spleen, and kidney, respectively, of control mouse; b, d, and f, liver, spleen, and kidney, respectively, of transgenic mouse. Blank and white arrows were used to point out the necrotic cells and fiber-like precipitation of protein, respectively.

transgenes to their offspring. This is consistent with previous reports that co-microinjection normally leads to the co-integration of two transgenes into a single chromosomal site [31,32].

The expression of sIgD showed that the gene copy number was not directly proportional to the level of gene expression. This is consistent with previous studies indicating that the transgene integration sites reflect gene expression levels better than does the copy number [33]. Deletion of a single *Mvk* allele in the mouse was able to increase sIgD 9–12-fold compared with negative control mice [14]. We inserted the target gene into the multiple cloning site of pcDNA3.1(+) to construct a high-expression sIgD transgenic mouse model, and expression of the transgenes could be detected by RT-PCR in the heart, liver, spleen, lung, kidney, intestine, brain, and muscle (data not shown). The lack of increase in serum sIgD may be for one of the three following reasons. First, the variable region of csIgD was derived from human rather than mouse IgG1, which may affect the spatial structure of the molecule and result in the affact of its identification by the antibody. Second, while we used the kappa light chain to construct csIgD, sIgD preferentially uses the lambda light chain [34,35], with the kappa light chain used for membrane IgD [36,37]. This might also affect the secretion and detection of csIgD. Finally, sIgD might have increased in serum but then have been rapidly degraded through a protective mechanism, making the increase undetectable. Further studies are needed to explain why we did not achieve stable and efficient expression of sIgD.

sIgD is a sign of early-stage B cell activation; it is also involved in the production and maintenance of memory B cells, and may play an important role in the sensitivity response of B cell tolerance. Therefore, if sIgD is increased, then B cell development is affected. Our flow cytometry analysis showed that there was no change in the proportion of B cells in the various stages of development (pro-B, pre-B, immature B, and mature B) between transgenic and control mice, indicating that the transgenes do not affect B cell development.

By genome walking, we demonstrated that the transgenes were integrated on different chromosomes in different transgenic mice, and that no functional genes (including *Mvk*) had been disrupted. This indicates that the inflammation in the transgenic mice is caused by increased sIgD rather than by *Mvk* mutation. sIgD has a proinflammatory function, and can effectively induce antimicrobial, immunostimulating, and proinflammatory factors such as IL-1β, TNF-α, IL-8, and CXCL10 [38]. Mucosal IgD can enhance local immunity by epithelial transport whereas circulating IgD binds to basophils through a calcium-mobilizing receptor. In addition to quickly mediating an innate immune response, IgD also conducts immune signaling when bacteria invade. When IgD-bound basophils encounter antigen, they migrate to the lymphoid system or mucosal immune lymphoid organ driven by chemokines that are released by mast cells under IgD stimulation. Basophils are organized through a calcium-mobilizing receptor that induces antimicrobial, opsonizing, inflammatory, and B cell-stimulating factors to enhance immune protection, and they induce an extreme immune response that leads to lesion destruction. Skin ulcers in transgenic mice with high expression of sIgD may be caused by excessive immune activation. In addition, displayed severe hepatosplenomegaly and renomegaly. These diseased organs had a high degree of cell necrosis and fiber-like protein precipitation—similar to states seen in human HIDS [5,22–25,39]. However, further studies are required to prove the usefulness of these transgenic mice as a model of HIDS.

*This work was supported by the National Basic Research Program of China (Grant No. 2010CB945300).*

1 Drenth J P, Prieur A M. Occurrence of arthritis in hyper-immunoglobulinaemia D. Ann Rheum Dis, 1993, 52: 765–766

2 Drenth J P, Boom B W, Toonstra J, *et al*. Cutaneous manifestations and histologic findings in the hyperimmunoglobulinemia D syndrome. International Hyper IgD Study Group. Arch Dermatol, 1994, 130: 59–65

3 Drenth J P, Haagsma C J, van der Meer J W. Hyperimmunoglobulinemia D and periodic fever syndrome. The clinical spectrum in a series of 50 patients. International Hyper-IgD Study Group. Medicine (Baltimore), 1994, 73: 133–144

4 van der Meer J W, Vossen J M, Radl J, *et al*. Hyperimmunoglobulinaemia D and periodic fever: a new syndrome. Lancet, 1984, 1: 1087–1090

5 Naruto T, Nakagishi Y, Mori M, *et al*. Hyper-IgD syndrome with novel mutation in a Japanese girl. Mod Rheumatol, 2009, 19: 96–99

6 Yoshimura K, Wakiguchi H. Hyperimmunoglobulinemia D syndrome successfully treated with a corticosteroid. Pediatr Int, 2002, 44: 326–327

7 Klasen I S, Goertz J H, van de Wiel G A, *et al*. Hyper-immunoglobulin A in the hyperimmunoglobulinemia D syndrome. Clin Diagn Lab Immun, 2001, 8: 58–61

8 Frenkel J, Rijkers G T, Mandey S H, *et al*. Lack of isoprenoid products raises *ex vivo* interleukin-1beta secretion in hyperimmunoglobulinemia D and periodic fever syndrome. Arthrit Rheumat, 2002, 46: 2794–2803

9 Drenth J P, Cuisset L, Grateau G, *et al*. Mutations in the gene encoding mevalonate kinase cause hyper-IgD and periodic fever syndrome. International Hyper-IgD Study Group. Nat Genet, 1999, 22: 178–181

10 Simon A, Cuisset L, Vincent M F, *et al*. Molecular analysis of the mevalonate kinase gene in a cohort of patients with the hyper-igd and periodic fever syndrome: its application as a diagnostic tool. Ann Intern Med, 2001, 135: 338–343

11 Houten S M, van Woerden C S, Wijburg F A, *et al*. Carrier frequency of the V377I (1129G>A) MVK mutation, associated with Hyper-IgD and periodic fever syndrome, in the Netherlands. Eur J Hum Genet, 2003, 11: 196–200

12 Aringer M. Periodic fever syndromes—a clinical overview. Acta Med Austriaca, 2004, 31: 8–12

13 Drenth J P, van der Meer J W. Hereditary periodic fever. N Engl J Med, 2001, 345: 1748–1757

14 Hager E J, Tse H M, Piganelli J D, *et al*. Deletion of a single mevalonate kinase (Mvk) allele yields a murine model of hyper-IgD syndrome. J Inherit Metab Dis, 2007, 30: 888–895

15 Ammouri W, Cuisset L, Rouaghe S, *et al*. Diagnostic value of serum immunoglobulinaemia D level in patients with a clinical suspicion of hyper IgD syndrome. Rheumatology, 2007, 46: 1597–1600

16 Dikeacou T C, van Joost T, Cormane R H. The recruitment of inflammatory cells using the skin-window technique. Arch Dermatol Res, 1979, 265: 1–7

17 Chen K, Cerutti A. New insights into the enigma of immunoglobulin D. Immunol Rev, 2010, 237: 160–179

18 Vladutiu A O. Immunoglobulin D: properties, measurement, and clinical relevance. Clin Diagn Lab Immun, 2000, 7: 131–140

19 Gorski K, Carneiro M, Schibler U. Tissue-specific in vitro transcription from the mouse albumin promoter. Cell, 1986, 47: 767–776

20 Herbomel P, Rollier A, Tronche F, *et al*. The rat albumin promoter is composed of six distinct positive elements within 130 nucleotides. Mol Cell Biol, 1989, 9: 4750–4758

21 Heard J M, Herbomel P, Ott M O, *et al*. Determinants of rat albumin promoter tissue specificity analyzed by an improved transient expression system. Mol Cell Biol, 1987, 7: 2425–2434

22 Kraus C L, Culican S M. Nummular keratopathy in a patient with Hyper-IgD Syndrome. Pediatr Rheumatol Online J, 2009, 7: 14

23 Sornsakrin M, Wenner K, Ganschow R. B cell cytopenia in two brothers with hyper-IgD and periodic fever syndrome. Eur J Pediatr, 2009, 168: 825–831

24 Attout H, Guez S, Ranaivo I, *et al*. A patient with hyper-IgD syndrome responding to simvastatin treatment. Eur J Intern Med, 2008, 19: e82–83

25 Coban E, Terzioglu E. A patient with hyper-IgD syndrome in Antalya, Turkey. Clin Rheumatol, 2004, 23: 177–178

26 Tronche F, Rollier A, Bach I, *et al*. The rat albumin promoter: cooperation with upstream elements is required when binding of APF/HNF1 to the proximal element is partially impaired by mutation or bacterial methylation. Mol Cell Biol, 1989, 9: 4759–4766

27 Zhang D E, Ge X, Rabek J P, *et al*. Functional analysis of the trans-acting factor binding sites of the mouse alpha-fetoprotein proximal promoter by site-directed mutagenesis. J Biol Chem, 1991, 266: 21179–21185

28 Izban M G, Papaconstantinou J. Cell-specific expression of mouse albumin promoter—evidence for cell-specific DNA elements within the proximal promoter region and cis-acting DNA elements upstream of -160. J Biol Chem, 1989, 264: 9171–9179

29 Pinkert C A, Ornitz D M, Brinster R L, *et al*. An albumin enhancer located 10-kb upstream functions along with its promoter to direct efficient, liver-specific expression in transgenic mice. Gene Dev, 1987, 1: 268–276

30 Hu J M, Camper S A, Tilghman S M, *et al*. Functional analyses of albumin expression in a series of hepatocyte cell lines and in primary hepatocytes. Cell Growth Differ, 1992, 3: 577–588

31 Clark A J, Cowper A, Wallace R, *et al*. Rescuing transgene expression by co-integration. Biotechnology (NY), 1992, 10: 1450–1454

32 Tang B, Yu S, Zheng M, *et al*. High level expression of a functional human/mouse chimeric anti-CD20 monoclonal antibody in milk of transgenic mice. Transgenic Res, 2008, 17: 727–732

33 Enjuanes L, Sola I, Castilla J, *et al*. Transgenic mice secreting coronavirus neutralizing antibodies into the milk. J Virology, 1998, 72: 3762–3772

34 Fine J M, Rivat C, Lambin P, *et al*. Monoclonal IgD. A comparative study of 60 sera with IgD “M” component. Biomedicine, 1974, 21: 119–125

35 Fibbe W E, Jansen J. Prognostic factors in IgD myeloma: a study of 21 cases. Scand J Haematol, 1984, 33: 471–475

36 Rowe D S, Hug K, Forni L, *et al*. Immunoglobulin-D as a lymphocyte receptor. J Exp Med, 1973, 138: 965–972

37 Ligthart G J, Schuit H R, Hijmans W. Subpopulations of mononuclear cells in ageing: expansion of the null cell compartment and decrease in the number of T and B cells in human blood. Immunology, 1985, 55: 15–21
38 Chen K, Xu W, Wilson M, *et al*. Immunoglobulin D enhances immune surveillance by activating antimicrobial, proinflammatory and B cell-stimulating programs in basophils. Nat Immunol, 2009, 10: 889–898
39 Obici L, Manno C, Muda A O, *et al*. First report of systemic reactive (AA) amyloidosis in a patient with the hyperimmunoglobulinemia D with periodic fever syndrome. Arthritis Rheum, 2004, 50: 2966–2969

**Open Access** This article is distributed under the terms of the Creative Commons Attribution License which permits any use, distribution, and reproduction in any medium, provided the original author(s) and source are credited.
